# Thermally Evaporated Copper Iodide Hole-Transporter for Stable CdS/CdTe Thin-Film Solar Cells

**DOI:** 10.3390/nano12142507

**Published:** 2022-07-21

**Authors:** Thuraisamykurukkal Thivakarasarma, Adikari Arachchige Isuru Lakmal, Buddhika Senarath Dassanayake, Dhayalan Velauthapillai, Punniamoorthy Ravirajan

**Affiliations:** 1 Clean Energy Research Laboratory, Department of Physics, University of Jaffna, Jaffna 40000, Sri Lanka; tsarma1990@gmail.com; 2 Faculty of Engineering and Science, Western Norway University of Applied Sciences, P.O. Box 7030, 5020 Bergen, Norway; 3 Postgraduate Institute of Science, University of Peradeniya, Peradeniya 20400, Sri Lanka; aaisurulakmal@gmail.com (A.A.I.L.); buddhikad@pdn.ac.lk (B.S.D.); 4 Department of Physics, University of Peradeniya, Peradeniya 20400, Sri Lanka

**Keywords:** CdS/CdTe thin-film solar cells, hole-transport layer, CuI, Cu diffusion

## Abstract

This study focuses on fabricating efficient CdS/CdTe thin-film solar cells with thermally evaporated cuprous iodide (CuI) as hole-transporting material (HTM) by replacing Cu back contact in conventional CdS/CdTe solar cells to avoid Cu diffusion. In this study, a simple thermal evaporation method was used for the CuI deposition. The current-voltage characteristic of devices with CuI films of thickness 5 nm to 30 nm was examined under illuminations of 100 mW/cm^2^ (1 sun) with an Air Mass (AM) of 1.5 filter. A CdS/CdTe solar cell device with thermally evaporated CuI/Au showed power conversion efficiency (PCE) of 6.92% with J_SC_, *V_OC_*, and FF of 21.98 mA/cm^2^, 0.64 V, and 0.49 under optimized fabrication conditions. Moreover, stability studies show that fabricated CdS/CdTe thin-film solar cells with CuI hole-transporters have better stability than CdS/CdTe thin-film solar cells with Cu/Au back contacts. The significant increase in FF and, hence, PCE, and the stability of CdS/CdTe solar cells with CuI, reveals that Cu diffusion could be avoided by replacing Cu with CuI, which provides good band alignment with CdTe, as confirmed by XPS. Such an electronic band structure alignment allows smooth hole transport from CdTe to CuI, which acts as an electron reflector. Hence, CuI is a promising alternative stable hole-transporter for CdS/CdTe thin-film solar cells that increases the PCE and stability.

## 1. Introduction

Cadmium telluride thin-film solar cells are one of the most cost-effective and reliable photovoltaic devices, with reported power conversion efficiencies over 22% [1]. Further, large-scale CdTe solar panels with efficiency over 18% are commercially available [2]. CdTe solar cells mainly contain a transparent conductive layer, n-CdS window layer, p-CdTe absorber layer and back contact. The power conversion of solar cells mainly depends on the thickness, morphology, and opto-electrical property of each layer. It can be controlled by the fabrication conditions of each layer. The conventional efficient CdS/CdTe solar cells have Cu back contact. Power conversion efficiency and stability of the conventional CdS/CdTe/Cu device not only depend on the fabrication conditions but also on copper diffusion to the CdS/CdTe interface. Chemical bath deposition (CBD), closed space sublimation (CSS), and thermal evaporation techniques are promising methods for depositing CdS, CdTe, and back contact layers. To avoid the Cu diffusion in these photovoltaic devices, Cu-free back contacts, such as wide bandgap metal oxides (NiO, MoO_3_, V_2_O_5_, and WO_3_), have been introduced as an alternative to Cu [3,4,5,6,7,8,9]. The electronic band alignment between CdTe and back contact directly affects the charge transport and recombination properties. Well-aligned band levels can improve the charge transport and reduce the recombination. A few organic materials, such as poly(3,4-ethylenedioxythiophene) doped with polystyrenesulfonate (PEDOT:PSS), robust cross-linkable conjugated polymer poly (diphenylsilane-co-4-vinyl-triphenylamine) Si-TPA, and P3HT, have also been employed as back contact buffer layers for CdTe thin-film solar cells [10,11,12]. However, the highest efficiency has only been achieved in CdS/CdTe solar cells with Cu back contact, despite the diffusion of Cu at the CdS/CdTe interface. The search for a better alternative to back contact motivated this study to consider CuI as a hole transport material (HTM).

Copper iodide has suitable properties that contribute to its application as an HTM in solar cells, such as a wide band gap (Eg) of 3.1 eV and stable p-type conductivity at room temperature. Depending on the thermal stability, the crystal structure of CuI is classified into α, β, and γ-structural phases. The α-phase is a cubic structure with a high temperature of 392 °C, the hexagonal β-phase is an ionic conductor with a temperature range of 350–392 °C, and the γ-phase is a cubic structure with a low temperature, below 350 °C [13]. Mainly, the optical and electrical properties of CuI can be tuned through the synthesis and preparation conditions. Several studies show the successful utilization of γ-CuI as a hole transport layer in solid-state dye-sensitized [14], organic solar cells [15] and perovskite solar cells [16,17,18,19,20,21,22,23,24], due to their band-matching and hole transporting properties. These show that a CuI hole-transporter not only enhances the power conversion efficiency but also improves charge extraction and stability. CuI has also been used as a hole selective contact in light-emitting diodes [25,26,27,28]. Moreover, CuI is economical and chemically stable and has high hole mobility, with a suitable energy level with CdTe and the back electrode.

CuI films are prepared using various techniques, such as solution process deposition (spray coating [29,30], spin coating [31,32]) [33], electro-deposition [34], chemical bath deposition [35], sputtering [36], pulse laser deposition [37], and thermal evaporation [38,39]. Each of these deposition methods has its own advantages and disadvantages. Solution process deposition and electro-deposition need a specialized precursor that limits their applications. CuI thin films, which are deposited using solution processes, are known to carry a high resistivity. Further, the deposition process leads to the yield of corrosive and toxic by-products [29,40,41,42]. Zhu et al. reported that CuI films grown with a pulse laser deposition (PLD) technique exhibited resistivity of 0.1–1 Ωcm and a transmittance of about 60–80% in a 410–1000 nm range [37]. Tanaka et al. obtained CuI films by thermal evaporation techniques with the same transmittance as Radio frequency-Direct current magnetron sputtering (RF-DC coupled magnetron sputtering), while the reported resistivity was 10^−2^ Ωcm [43]. Compared to previous reports, the resistivity obtained in this work was too high to make it widely usable in LEDs. CuI layers with low resistivity and high transmittance have been used in various solar cells as hole-transporters. During the sputtering deposition, the direct use of nanoparticles can lead to agglomeration behavior and, thus, form an unsmooth layer. For the hole transporting purpose, it is necessary to prepare low-resistive CuI.

Thermal evaporation has many advantages, such as being non-pollutant, easy to control the deposition rate and film thickness, and suitable for preparing larger area fabrication with smooth surfaces and high chemical purities. Therefore, the thermal evaporation method is more suitable for preparing γ-phase CuI films with high conductivity and high hole mobility [38,43]. This study focuses on thermally evaporated CuI as an alternative hole-transporter in CdS/CdTe thin-film solar cells.

## 2. Materials and Methods

The substrates or films were kept in an open environment except during fabrication. The CdS/CdTe thin-film solar cells were fabricated with structure of glass/SnO_2_:F(FTO)/n-CdS/p-CdTe/CuI/Au. FTO-coated substrates (Sigma-Aldrich, St. Louis, MI, USA) were cleaned as reported elsewhere [44]. Window n-CdS layer of 80 nm thickness was deposited on cleaned FTO substrates by the chemical bath deposition technique (CBD) at 90 °C as reported elsewhere [44,45,46,47]. The CBD reaction solution contains de-ionized water, cadmium acetate, ammonium acetate, and thiourea. The CdS-coated film was ultrasonically washed to remove loosely bound CdS particles on the surface. Before further device processing began, CdS was etched off the glass side of the samples with a dilute HCl solution. After air drying, heat treatment for the CdS layer was conducted in a N_2_ environment at 375 °C for 30 min. Thereafter, ~5 μm CdTe absorber layer was deposited on CBD-CdS by close-spaced sublimation (CSS) system (MTI, Richmond, VA, USA), with source and substrate temperatures of 640 °C and 580 °C, respectively, under various chamber pressures of 5.0 Torr and 7.9 torr in an Argon medium to investigate the effect of CSS conditions for CdTe layer. In order to investigate the effect of a CuI hole transport layer on CdTe solar cells, CuI films with thicknesses ranging from 5 nm to 30 nm were deposited on the CdTe film by thermal evaporation (Edwards, West Sussex, UK). Following the CuI film deposition, an ~80 nm thick Au layer was thermally evaporated. CBD-CdS/CSS-CdTe/Cu/Au devices were also fabricated as a comparison to demonstrate beneficial effects induced by a CuI hole-transporter. All the fabricated devices were later annealed at 200 °C for 10 min in N_2_ environment.

The electrical properties of CuI were measured using the four-point probe (FPP) technique (SES Instruments DFP-03, Uttarakhand, India). The optical and structural properties of each layer were characterized by using UV-Vis (JENWAY-6800, Stone, UK), XRD (PANalytical-AERIS, Eindhoven, The Netherlands) spectroscopy, and Atomic Force Microscopy (Park systems-XE7, Suwon-si, Korea). XPS analysis was performed using the Thermo Scientific ESCALAB Xi instrument (Thermo Fisher Scientific, Waltham, MA, USA) with Al Kα as the X-ray source. Finally, their photovoltaic performance and device stability were analyzed using a solar simulator (PEC-L12, Yokohama, Japan) under the illumination of 100 mW/cm^2^ with an Air Mass of 1.5 filter.

## 3. Results and Discussion

### 3.1. Structural Characterization

**Figure 1:**
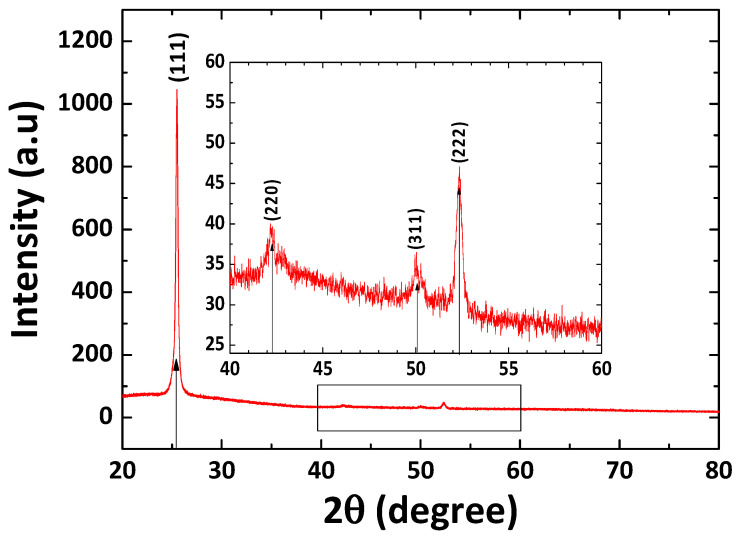
XRD pattern of thermally evaporated CuI film.

Figure 1 shows the XRD pattern of the CuI thin film with characteristic peaks at the 2θ values of 25.5°, 42.2°, 50.0°, and 52.4° that coincide with the (111), (220), (311), and (222) atomic plane of the zinc blend face centered cubic γ-phase CuI structure, respectively, and values agree with JCPDS card No.06-0246 [17,43]. Therefore, it is confirmed that the CuI thin film is a hole transport semiconductor, since it is a γ-phase rather than the ionic conductor of the α or β phase [17]. The average crystalline size of the the thermally evaporated CuI film was estimated using the predominant (111) plane by the Debye–Scherrer equation:

(1) $$d=\frac{k\lambda }{\beta \cos\theta }$$

where d is the average crystallite size, k is the dimensionless shape factor, which has a typical value of about 0.89, λ is the wavelength of the X-ray beam (0.5406 nm), θ is the Bragg angle, and β is the full width at half maxima (FWHM). The calculated crystallite size was about 25.35 nm.

**Figure 2:**
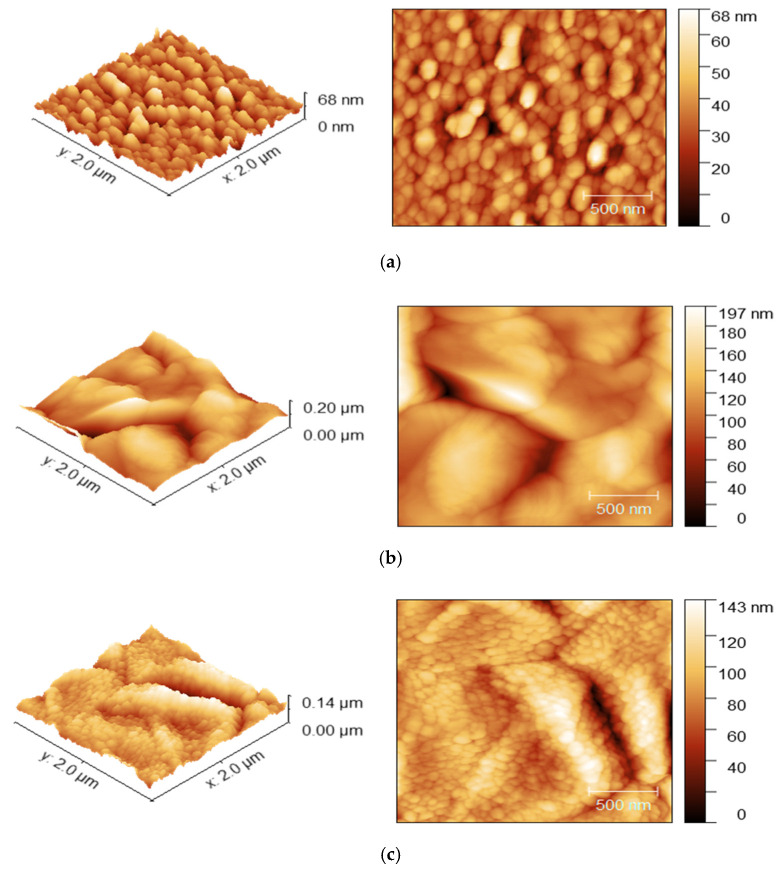
3D and 2D AFM images of (**a**) CBD-CdS; (**b**) CdS/CSS-CdTe, and (**c**) CdS/CdTe/TE-CuI film.

Figure 2 shows the topography images of CBD-CdS, CSS-CdTe, and thermally evaporated CuI on the CdS/CdTe film with a scanning area of 2.0 μm × 2.0 μm. These confirm the uniformity of each CBD-CdS, CSS-CdTe, and CuI on the CdTe films and no pinholes in layers were observed from AFM analysis. The average and Root mean square (RMS) roughness values are tabulated in Table 1. The RMS roughness is slightly decreased from 25.32 nm to 18.72 nm, indicating a smooth coating of CuI particles. Similar topographical changes were observed by Deng-Bing Li et.al. [48].

The chemical-bath-deposited CdS surface has low roughness of 33.60 nm (RMS of 8.42 nm). This was further evidence for higher transmission of the CdS layer due to the lower light scattering. As the roughness of the CdS window layer, as shown in Figure 2a, is low, it makes an ideal substrate for the CSS- CdTe absorber layer. The closed-space sublimated CdTe film, as shown in Figure 2b, has a roughness of 114.30 nm (RMS of 25.32 nm). The grain size of the CdTe layer also makes it an ideal substrate for the CuI layer and thermally evaporated CuI on the CdTe surface, as shown in Figure 2c, has a roughness of 87 nm (RMS of 18.72 nm). Since thermally evaporated CuI particles uniformly covered the CdTe surface without any pinholes, it provides good electrical contact with Au.

### 3.2. Opto-Electrical Characterization

**Figure 3:**
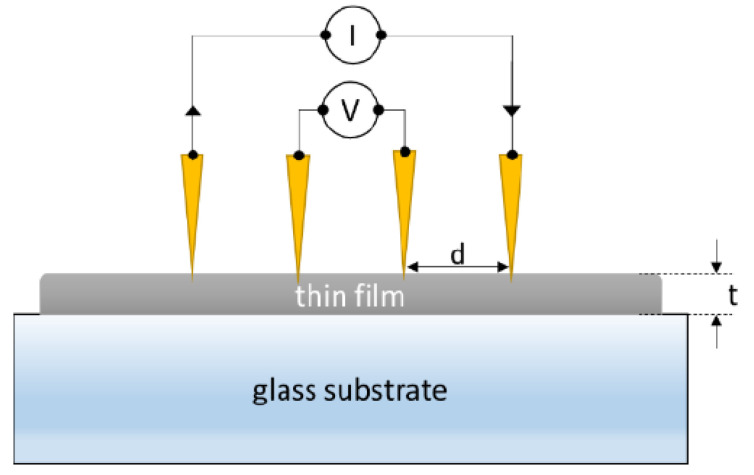
Schematic diagram of the four-point probe technique.

The electrical resistivity of the thermally evaporated CuI film was measured by using the four-point probe measurement technique (FPP) [49], which is shown schematically in Figure 3. To perform the measurement, a DC current I was passed through the outer probes and the potential difference V between the inner probes was measured.

When the probe distance d is much larger than the film thickness, t, sheet resistance, $R_{S}$ (in Ω/sq), and resistivity, ρ (in Ωcm), can be determined from Equations (2) and (3):

(2) $$R_{S}=\frac{\pi }{ln2}\frac{V}{I}\approx 4.53\frac{V}{I}$$

and

(3) $$\rho =R_{S}.t$$

The sheet resistance and resistivity of fabricated CuI were measured as 19.856 kΩ/sq and 0.20 Ωcm, respectively (Figure S2), at room temperature, which agrees with the literature value of thermally evaporated CuI films [38].

**Figure 4:**
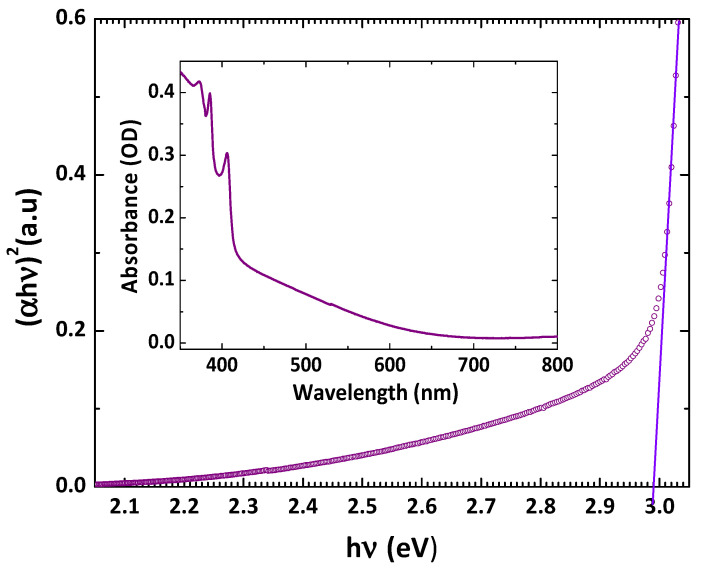
Tauc plot and absorption spectrum (inset) of thermally evaporated CuI film.

Figure 4 shows the Tauc plot of ${\left(\alpha h\nu \right)}^{2}$ versus hν of the thermally evaporated CuI film, where α is the absorption coefficient, and h and ν are the Planck constant and photon frequency, respectively. The inset shows the absorption spectrum of the thermally evaporated CuI film. The bandgap of the CuI thin film can be calculated by Equation (4).

(4) $$\alpha h\nu =A{\left(h\nu -E_{g}\right)}^{1/2}$$

where A is a parameter related to the electronic band structure, carrier effective mass, and the refractive index of the material and $E_{g}$ is the bandgap energy. The obtained bandgap of the CuI film was 2.99 eV, which is in agreement with the reported value of ~3.0 eV [38]. The bandgaps of CBD-CdS and CSS-CdTe were 2.35 and 1.48 eV, respectively (Figure S1). The obtained values are in agreement with the reported values of CBD-CdS [50,51,52] and CSS-CdTe [53,54].

**Figure 5:**
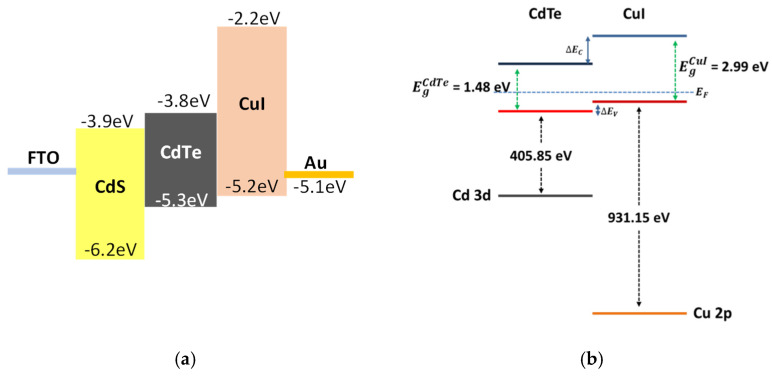
(**a**) Proposed energy alignment of FTO/CdS/CdTe/CuI/Au interface and (**b**) the quantitative electronic band alignment at the CSS-CdTe/TE-CuI/TE-Au interface obtained from XPS.

The band alignment of the CdTe/CuI interface is important to the hole transport and formation of low electronic resistance, as it enhances the hole transport and reduces carrier recombination. Figure 5 shows the proposed energy levels of CuI, which match with CdS/CdTe energy levels of calculated values of the valance band and conduction band level derived from XPS data. The values for the VBMs were obtained as 0.5 eV and 0.25 eV for CdTe and CuI, respectively, from XPS spectra of bulk CdTe and bulk CuI [55,56].

**Figure 6:**
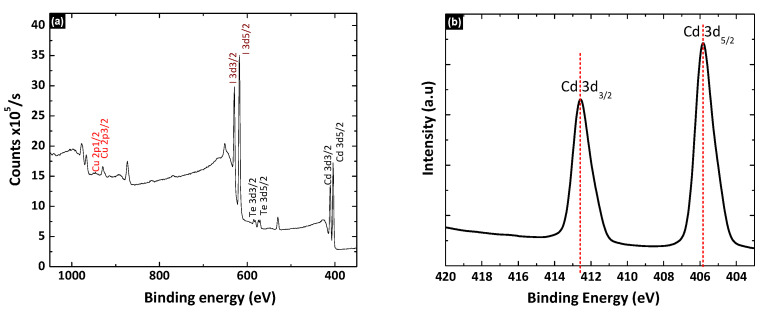

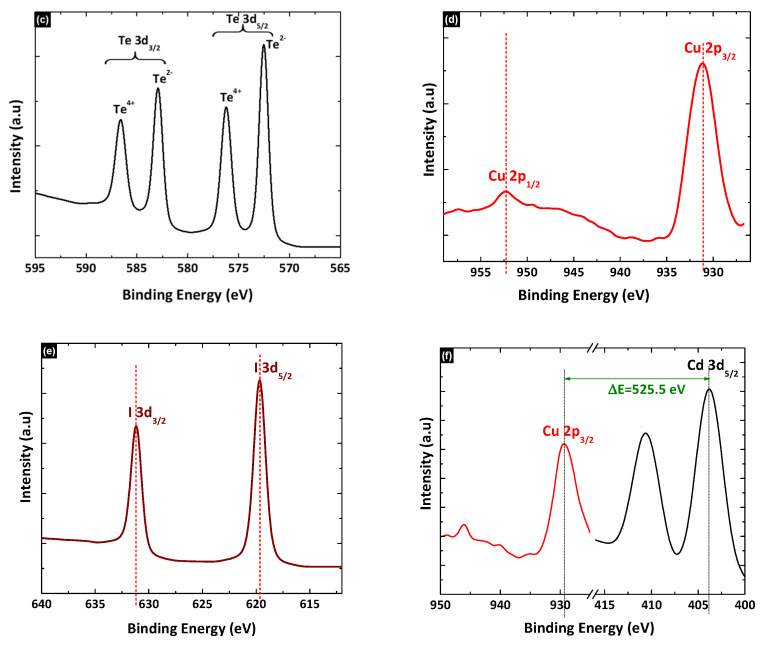
(**a**) XPS survey spectrum of CdTe/CuI interface, (**b**) core level of the Cd 3d region, (**c**) core level of the Te 3d region, (**d**) core level of the Cu 2p region, (**e**) core level of the I 3d region, and (**f**) high-resolution spectra of the Cd 3d and Cu 2p regions in CdTe/CuI interface.

Figure 6a, shows the XPS spectra of the thermally evaporated CuI film on the CdTe layer. This XPS confirms the stable interface formation without a shift in the binding energy of either Cu or I. The XPS peaks of core level Cd were observed at 412.55 eV and 405.85 eV, corresponding to the 3d 3/2 and 3d 5/2 transition (Figure 6b). Similarly, the XPS peaks of core level Te were observed at 582.95 eV and 572.55 eV corresponding to the Te 3d 3/2 and 3d 5/2 transition (Figure 6c). Figure 6d shows the XPS peaks for Cu were observed at 952.3 eV and 931.15 eV corresponding to the 2p1/2 and 2p3/2 transition, respectively. Similarly, Figure 6d shows the XPS peaks of I, which were observed at 631.2 eV and 619.65 eV, corresponding to 3d3/2 and 3d5/2 transitions, respectively. The overall chemical structure of the CdTe/CuI was found to be significantly stable. At the CdTe/CuI interface, the energy difference between the Cd 3d5/2 and the Cu 2p3/2 core levels (Figure 6f) was 525.5 eV.

Based on the XPS measurements, the valance band offset value of $\Delta E_{V}$ at the CdTe/CuI interface was calculated to be 0.25 eV. Further, the conduction band offset $\Delta E_{C}$ at the CdTe/CuI interface can be found from Equation (5):

(5) $$\Delta E_{C}=\Delta E_{V}+E_{g}^{CuI}-E_{g}^{CdTe}$$

where $E_{g}^{CuI}$ and $E_{g}^{CdTe}$ are the optical band gap values of CuI and CdTe, respectively. The calculated $\Delta E_{C}$ was 1.76 eV. The small valence band offset between CdTe and CuI ($\Delta E_{V}$) enhances the hole transport from CSS-CdTe to TE-CuI without any barrier. The large conduction band offset between CdTe and CuI ($\Delta E_{C}$) with a value of 1.76 eV indicates that the CuI can act as an electron-blocking layer, which helps repel electron transport from the Au back contact and also reduce carrier recombination. The chemical structure of CdTe was preserved, even after CuI deposition, confirming the effectiveness of the fabrication process of CdTe and CuI layers.

### 3.3. Current-Voltage (J–V) Characteristics of CdS/CdTe Thin-Film Solar Cells

**Figure 7:**
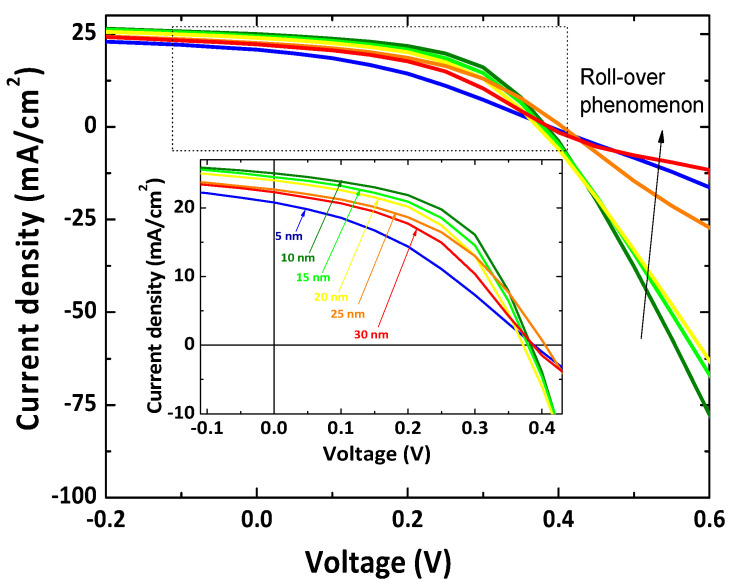
J–V characteristic curves of CBD-CdS/CSS-CdTe solar cells (without CdCl_2_ treatment) with CuI with thicknesses of 5, 10, 15, 20, 25, and 30 nm.

**Figure 8:**
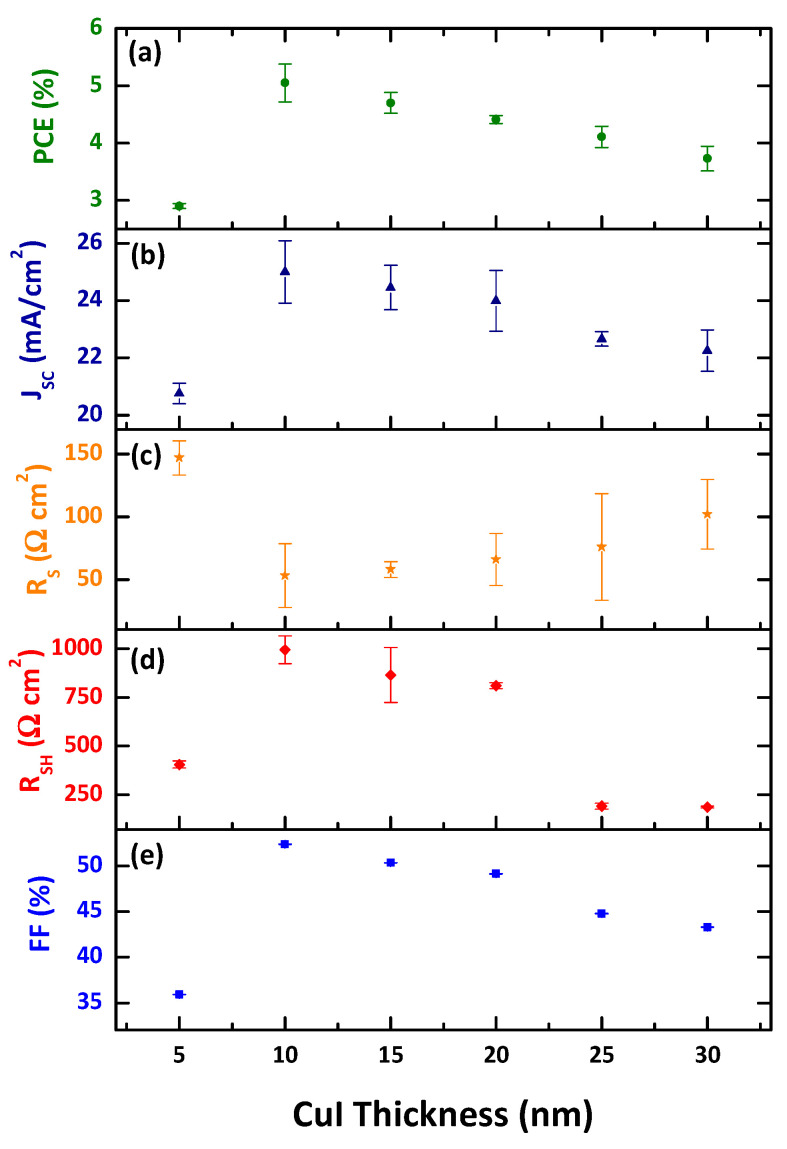
Photovoltaic parameters of (**a**) power conversion efficiency, (**b**) short circuit current density, (**c**) series resistance, (**d**) shunt resistance, and (**e**) fill factor of the CBD-CdS/CSS-CdTe devices with different CuI thicknesses.

The current-voltage characteristic curves of CBD-CdS/CSS-CdTe solar cells with different thicknesses of the CuI hole transport layer are shown in Figure 7. The figure clearly indicates that the thermally evaporated CuI thickness can remarkably influence CdS/CdTe device performance. The corresponding photovoltaic parameters are summarized in Figure 8. It is found that the roll-over phenomenon in J–V characteristics was less prominent when the thickness of the hole-transporter was reduced, as reported elsewhere [57]. In the literature, the roll-over is most frequently explained by the back contact barrier [58,59,60,61] and the photo-conducting properties of the n-CdS window layer [62,63].

The short circuit current density is influenced by the series resistance (R_S_), which increases with CuI thickness due to the high resistivity in the thicker CuI layer preventing the carrier transport. The devices with 10nm of CuI thickness deliver the best performance due to the lowest R_S_ and largely improved J_SC_, R_SH,_ and FF, compared to those devices with lower and higher thicknesses. This is mainly due to the higher hole concentration in CdTe. Higher hole concentration can boost a higher build-in potential (V_bi_) and lead to less recombination in the depletion region and at the front interface, thereby resulting in larger FF, which can be further confirmed through an improvement in the shunt resistance (R_SH_). Roussillon et al. also suggested that the back contact barrier on the properties of CdTe solar cells depend on the space charge region in the main CBD-CdS/CSS-CdTe junction and the space charge region in the back contact may overlap, depending on the barrier height of the back contact [64]. Thus, the changed band diagram affects the collection and recombination of the carriers significantly. Accompanied by the variation in the CuI thickness, the shunt resistance gradually increases from 404 Ωcm^2^ for 5 nm to 994 Ωcm^2^ for 10 nm thickness of CuI. When the thickness of CuI was further increased to 30 nm, the R_SH_ decreases to 186 Ωcm^2^. The J_SC_ also shows a similar trend as the FF and the shunt resistance with the CuI hole-transport layer thickness. A similar trend was observed in a reported study [65].

**Figure 9:**
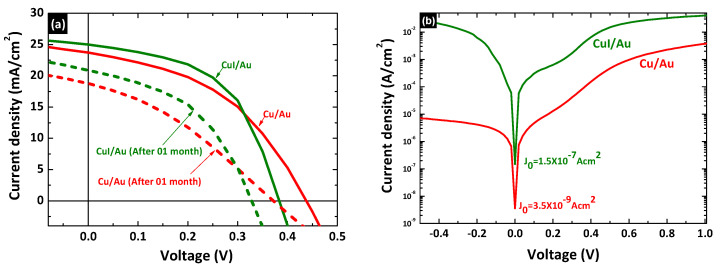
J–V curves of CBD-CdS/CSS-CdTe solar cells with a Cu/Au and CuI/Au back contact (**a**) under illumination of 100 mW/cm^2^ with AM1.5 filter and (**b**) the semi-logarithmic J–V characteristics of CBD-CdS/CSS-CdTe solar cells with Cu/Au and CuI/Au back contact in dark.

The measured photovoltaic performances of the fabricated devices instantly after fabrication and after one month for devices with 10 nm Cu/Au bi-metal and CuI/Au back contacts are shown in Figure 9 and Table 2. Insertion of a CuI hole transport layer instead of Cu demonstrated a significant increase in the PCE due to improvement in the J_SC_, FF, and R_SH_. However, *V_OC_* in the device with Cu/Au is higher than that in the device with CuI/Au back contact due to carrier density increment in the CdTe absorber by involving Cu_X_Te through the diffusion of Cu, which is clearly described in previous studies [66,67,68]. *V_OC_* is given by Equation (6) [69] and can be confirmed by the dark J–V characteristic curve shown in Figure 9b.

(6) $$V_{OC}=\frac{nkT}{q}\ln\left(\frac{J_{SC}}{J_{0}}-1\right)$$

where $J_{0}$ is the saturation current density, $J_{SC}$ is the short-circuit current density, n is the diode ideality factor, q is the electronic charge, while k and T are Boltzmann constant and absolute temperature, respectively. CBD-CdS/CSS-CdTe devices with Cu/Au and CuI/Au back contacts exhibit dark saturation current densities of 3.5 × 10^−9^ A/cm^2^ and 1.5 × 10^−7^ A/cm^2^, respectively. This means the dark saturation current density in the device with Cu/Au back contact is nearly two orders of magnitude lower than that of the device with CuI/Au back contact. The Cu_X_Te structure is formed at the back of the CdTe absorber by the diffusion of copper, as mentioned earlier. Most of the Cu diffused into the bulk finds its way to the CdS with time, lowering its space charge density and causing it to become photoconductive. This effect of Cu in the CdTe is more problematic, adding recombination centers (lowering the efficiency) and increasing the space charge density, which might either raise or lower efficiency, depending on CdTe thickness [61]. This is why power conversion efficiency drops by ~50% within a month due to all photovoltaic parameter losses, as described in Table 2. The J_SC_ of the device with a CuI hole-transporter was significantly higher than the device with Cu/Au back contacts, which is due to the hole transport property of the thermally evaporated CuI and band alignment between the CdTe and CuI. The fill factor of the devices with a CuI/Au was significantly higher than that of the Cu/Au devices, which was induced by the smaller R_S_ and larger R_SH_ value of the device with a CuI/Au back contact than Cu/Au back contact.

### 3.4. Influence of Fabrication Conditions on J–V Characteristics of CdS/CdTe/CuI/Au Device

**Figure 10:**
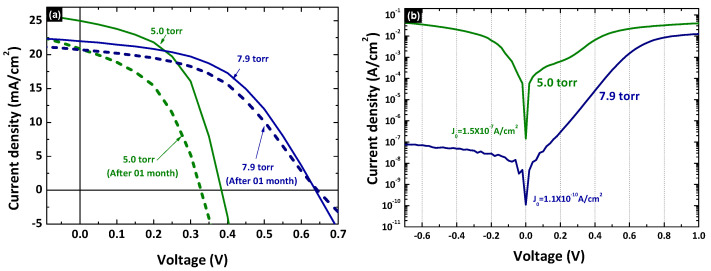
(**a**) J–V characteristic of CdS/CdTe/CuI/Au device fabricated with different CSS parameter under illumination of 100 mW/cm^2^ with AM1.5 filter and (**b**) semi-logarithmic plot in dark.

The influence of different growth conditions of CSS-CdTe on the photovoltaic performance of the device was also analyzed with CSS-CdTe growth conditions of 5.0 torr and 7.9 torr, which were fabricated with a 10 nm CuI hole-transporter. Figure 10 shows the corresponding J–V characteristic curve under the illumination of 100 mW/cm^2^ with AM 1.5 filter and in dark. Table 3, illustrates the photovoltaic parameter of the corresponding J–V characteristic curve.

**Figure 11:**
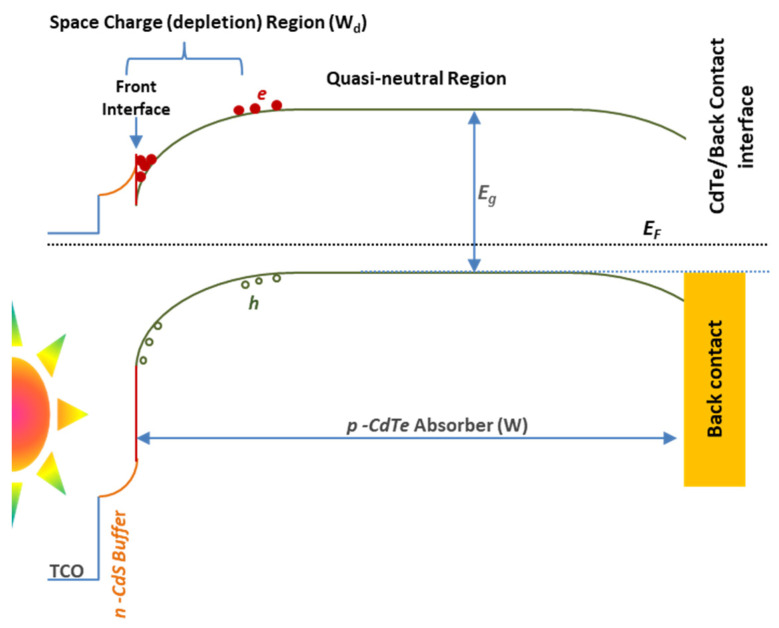
Energy band diagram of a generic FTO/n-CdS/p-CdTe/back-contact solar cell with the front interface, the depletion region, quasi-neutral region, and the back-contact interface.

This also shows the roll-over phenomenon disappeared in CdTe solar cells fabricated under 7.9 torr. Further, a later crossover of the light and dark J–V curves for the cell fabricated under 7.9 torr demonstrates that the energy barrier at the back contact was reduced, and the estimated reverse saturation diode current (J_0_) was $1.11\times 10^{-10}A/{\mathrm{cm}}^{2}$. n-CdS/p-CdTe/CuI/Au device growth condition of 7.9 torr exhibits higher power conversion efficiency of 6.92% with J_SC_, *V_OC_*_,_ and FF of 21.98 mA/cm^2^, 0.638 V and 0.49, respectively. *V_OC_* and FF in a generic FTO/CBD-CdS/CSS-CdTe/back-contact thin-film solar cell device is a key parameter in the recombination analysis. In particular, *V_OC_* is sensitively influenced by the interface recombination at the buffer/absorber, front interface, and the absorber/back-contact interface [70], as shown in Figure 11.

The highest *V_OC_* and FF illustrate the fewer recombination losses. Mathematically, *V_OC_* can be explained with recombination coefficients by Equation (7).

(7) $$V_{OC}=\frac{2k_{B}T}{q}ln\left[\frac{1}{2}\frac{R_{0}^{d}}{\left(R_{0}^{i,f}+R_{0}^{b}+R_{0}^{i,b}\right)}\left(\sqrt{G_{a}\frac{4W\left(R_{0}^{i,f}+R_{0}^{b}+R_{0}^{i,b}\right)}{{\left(R_{0}^{d}\right)}^{2}}+1}-1\right)\right]$$

where $R_{0}^{i,f}$, $R_{0}^{d}$, $R_{0}^{b}$, and $R_{0}^{i,b}$ are bias-independent (*V* = 0) recombination coefficients at the buffer/absorber interface, in the depletion region (W_d_), in the quasi-neutral region, and at the absorber/back-contact interface, respectively [70]. This means *V_OC_* depends on the carrier recombination of the solar cells influenced by the fabrication conditions of each layer. Fabrication conditions affected the grain size (Figure S4 and Table S2), surface roughness, and thickness of the layer. This was clearly discussed in several studies [54,71,72,73,74,75,76]. In this study, it can be seen in the Supporting Information. The CuI hole-transporter provides fewer recombination losses in CdS/CdTe thin-film solar cells. Further, 7.9 torr vacuum gives 40% efficiency enhancement in solar cells fabricated compared to 5.0 torr vacuum. For the device stability, photovoltaic performance was also tested after one month (Figure 10). Only less than 10% PCE drops within a month from 6.92% to 6.25% (Figure S5). This shows that the stability of the device not only depends on the hole-transporter but the fabrication condition of each layer also has a significant role.

## 4. Conclusions

Thermally evaporated CuI was successfully employed as a hole-transporter for CBD-CdS/CSS-CdTe solar cells. A significant improvement in fill-factor and, hence, efficiency was achieved by replacing Cu as back contact with CuI. Structural and opto-electrical properties of thermally evaporated CuI reveal that the deposited film has γ-phase CuI, which has good electrical conductivity and well-matched energy band alignment with CSS-CdTe. Further, XPS studies of the CdTe/CuI interface confirmed that a small valence band offset at the CdTe/CuI interface improves the photo-generated hole transport from CdTe to CuI without any barrier, and the large conduction band offset between CdTe and CuI reduces the electron transport from back contact to CdTe; thus, the insertion of CuI as a hole-transporter can reduce the electron recombination rate at the back contact. The results clearly showed that CuI is a promising hole-transporter instead of Cu as back contact for CdS/CdTe thin-film solar cells, as that will prevent Cu diffusion, increase stability, and also increase the PCE considerably.

## Figures and Tables

**Table 1:** Average and RMS roughness of each film calculated from AFM topography.

Film	Average Roughness (nm)	RMS Roughness (nm)
CBD-CdS	33.60	8.42
CSS-CdTe	114.30	25.32
TE-CuI	87.00	18.72

**Table 2:** Photovoltaic parameter measured instant after fabrication and after one month of CdS/CdTe devices with CuI/Au and Cu/Au back contacts.

Device	J_SC_ (mA/cm^2^)	V_OC_ (V)	FF	PCE (%)
**Cu/Au**	**23.71**	**0.44**	**0.44**	**4.57**
**Cu/Au After 01 month**	**18.78**	**0.37**	**0.34**	**2.35**
**CuI/Au**	**25.00**	**0.39**	**0.52**	**5.05**
**CuI/Au After 01 month**	**20.87**	**0.33**	**0.46**	**3.13**

**Table 3:** Photovoltaic parameter of CdS/CdTe/CuI/Au devices with different deposition conditions of CSS-CdTe.

CSS Condition	J_SC_ (mA/cm^2^)	V_OC_ (V)	FF	PCE (%)	PCE Drops
**5.0 torr**	**25.00**	**0.39**	**0.52**	**5.05**	**38%**
**After 01 month**	**20.87**	**0.33**	**0.46**	**3.13**	
**7.9 torr**	**21.90**	**0.63**	**0.49**	**6.92**	**10%**
**After 01 month**	**20.72**	**0.64**	**0.47**	**6.25**	

## Data Availability

The data in this paper are available in the Supplementary Materials.

## Supplementary Materials

The following supporting information can be downloaded at: https://www.mdpi.com/article/10.3390/nano12142507/s1, Figure S1: Optical absorption spectra of (a) chemically deposited CdS film and closed-space sublimated CdTe film; Figure S2: Temperature-dependent electrical property of thermally evaporated CuI; Figure S3: XRD pattern of (a) CBD-CdS and (b) CSS-CdTe; Figure S4: XRD pattern and AFM Topography images of CSS-CdTe with different fabrication parameters; Figure S5: (a) J–V characteristic and (b) variation in photovoltaic parameters of CdS/CdTe/CuI/Au solar cells with time; Table S1: Average crystallite size of CBD-CdS and CSS-CdTe; Table S2: Structural parameters of CSS-CdTe with different fabrication parameters.
