# Supplementary material for: Thermally Evaporated Copper Iodide Hole-Transporter for Stable CdS/CdTe Thin-Film Solar Cells
Source: Nanomaterials (Basel). 2022 Jul 21;12(14):2507. doi: 10.3390/nano12142507 (PMC9315675; doi:10.3390/nano12142507)
Supplement: Supplementary file 1 [file nanomaterials-12-02507-s001.zip › nanomaterials-1779491-supplementary.pdf]

Supporting Information

Thermally Evaporated Copper Iodide Hole-Transporter for Stable CdS/CdTe Thin-Film Solar Cells

Thuraisamykurukkal Thivakarasarma [1,2], Adikari Arachchige Isuru Lakmal [3,4], Buddhika Senarath Dassanayake [3,4], Dhayalan Velauthapillai [2,*] and Punniamoorthy Ravirajan [1,*]

[1] Clean Energy Research Laboratory, Department of Physics, University of Jaffna, Jaffna 40000, Sri Lanka; tsarma1990@gmail.com
[2] Faculty of Engineering and Science, Western Norway University of Applied Sciences, P.O. Box 7030, 5020 Bergen, Norway
[3] Postgraduate Institute of Science, University of Peradeniya, Peradeniya 20400, Sri Lanka; aaisurulakmal@gmail.com (A.A.I.L.); buddhikad@pdn.ac.lk (B.S.D.)
[4] Department of Physics, University of Peradeniya, Peradeniya 20400, Sri Lanka
* Correspondence: pravirajan@univ.jfn.ac.lk (P.R.); dhayalan.velauthapillai@hvl.no (D.V.)

(a) **(b)**

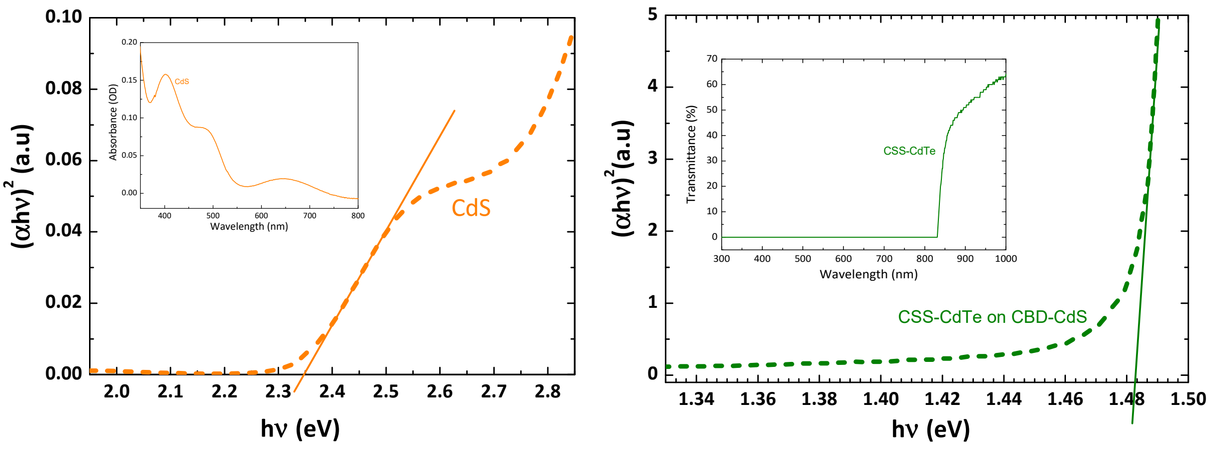

Figure S1: Optical absorption spectra of (**a**) Chemically deposited CdS film and (**b**) closed space sublimated CdTe film.

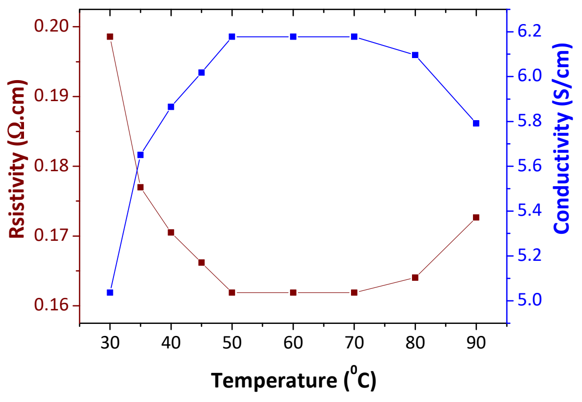

Figure S2: Temperature dependant electrical property of thermally evaporated CuI.

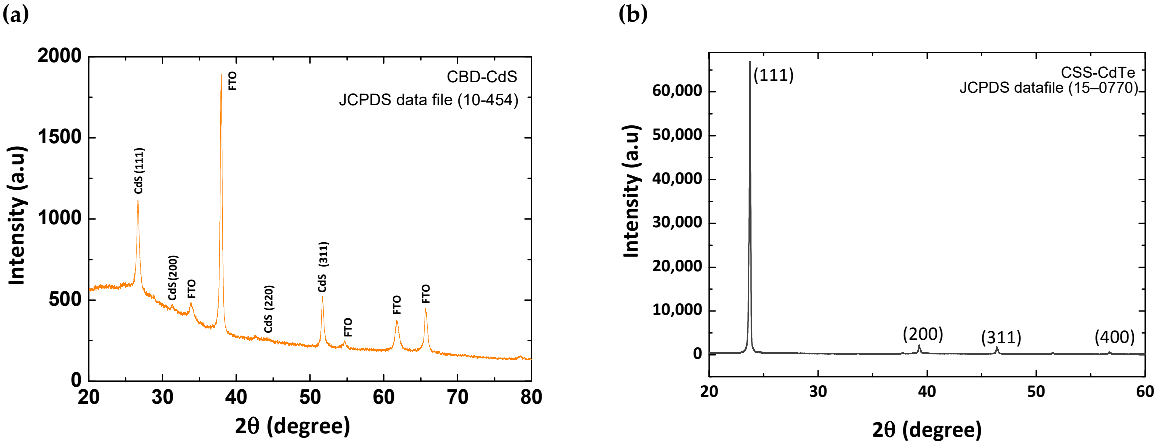

Figure S3: XRD pattern of (**a**) CBD-CdS and (**b**) CSS-CdTe.

Table S1: Average crystallite size of CBD-CdS and CSS-CdTe.

Film	Average crystallite size (nm)
CBD-CdS	18.20
CSS-CdTe	45.47

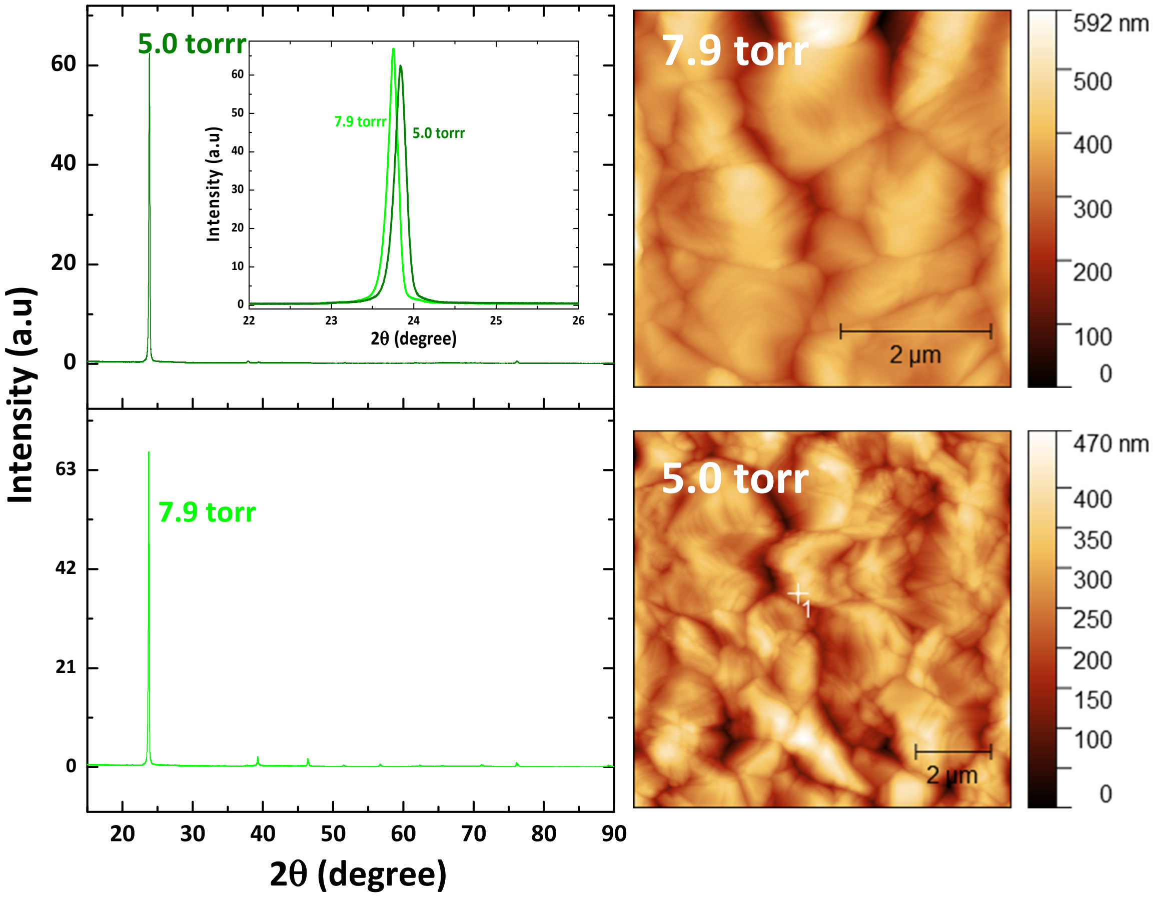

Figure S4: XRD pattern and AFM Topography images of CSS-CdTe with different fabrication parameter

Table S2: Structural parameters of CSS-CdTe with different fabrication parameter.

Vacuum Pressure	Lattice Parameter (Å)	Crystallite size D (nm)	Micro strain Ɛ (10^{-3})
7.9 torr	6.49	50.62	3.33
5.0 torr	6.46	45.47	3.69

(a) **(b)**

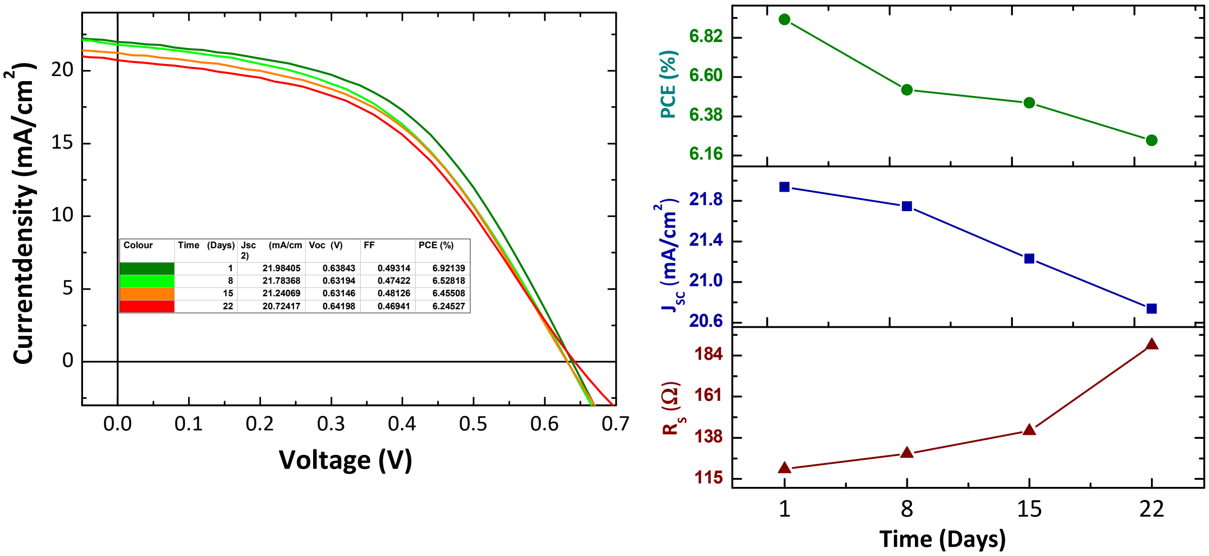

Figure S5: (a) J-V characteristic and (**b**) Variation of photovoltaic parameters of CdS/CdTe/CuI/Au solar cells with time.
